# Physical mapping and candidate gene prediction of fertility restorer gene of cytoplasmic male sterility in cotton

**DOI:** 10.1186/s12864-017-4406-y

**Published:** 2018-01-02

**Authors:** Cunpeng Zhao, Guiyuan Zhao, Zhao Geng, Zhaoxiao Wang, Kaihui Wang, Suen Liu, Hanshuang Zhang, Baosheng Guo, Junyi Geng

**Affiliations:** Institute of Cotton, Hebei Academy of Agriculture and Forestry Sciences, Key Laboratory of Biology and Genetic Improvement of Cotton in Huanghuaihai Semiarid Area, The Ministry of Agriculture, No.598 Heping west, Shijiazhuang, Hebei 050051 China

**Keywords:** CMS, High-throughput sequencing, SLAF-seq, Super-BSA, Cotton

## Abstract

**Background:**

Cytoplasmic male sterility (CMS) is a maternally inherited trait failing to produce functional pollen. It plays a pivotal role in the exploitation of crop heterosis. The specific locus amplified fragment sequencing (SLAF-seq) as a high-resolution strategy for the identification of new SNPs on a large-scale is gradually applied for functional gene mining. The current study combined the bulked segregant analysis (BSA) with SLAF-seq to identify the candidate genes associated with fertility restorer gene (*Rf*) in CMS cotton.

**Methods:**

Illumina sequencing systematically investigated the parents. A segregating population comprising of 30 + 30 F_2_ individuals was developed using 3096A (female parent) as sterile and 866R (male parent) as a restorer. The original data obtained by dual-index sequencing were analyzed to obtain the reads of each sample that were compared to the reference genome in order to identify the SLAF tag with a polymorphism in parent lines and the SNP with read-associated coverage. Based on SLAF tags, SNP-index analysis, Euclidean distance (ED) correlation analysis, and whole genome resequencing, the hot regions were annotated.

**Results:**

A total of 165,007 high-quality SLAF tags, with an average depth of 47.90× in the parents and 50.78× in F_2_ individuals, were sequenced. In addition, a total of 137,741 SNPs were detected: 113,311 and 98,861 SNPs in the male and female parent, respectively. A correlation analysis by SNP-index and ED initially located the candidate gene on 1.35 Mb of chrD05, and 20 candidate genes were identified. These genes were involved in genetic variations, single base mutations, insertions, and deletions. Moreover, 42 InDel markers of the whole genome resequencing were also detected.

**Conclusions:**

In this study, associated markers identified by super-BSA could accelerate the study of CMS in cotton, and as well as in other crops. Some of the 20 genes’ preliminary characteristics provided useful information for further studies on CMS crops.

**Electronic supplementary material:**

The online version of this article (10.1186/s12864-017-4406-y) contains supplementary material, which is available to authorized users.

## Background

As a maternally inherited characteristic, cytoplasmic male sterility (CMS) plays a major role in crop heterosis research and practice. The current studies suggest that CMS is caused by mutations in the correlated genes in the mitochondrial genome and inhibited by fertility restorer genes in the nuclear genome [1]. This phenomenon exists in bean [2, 3], petunia [4], sorghum [5], and rice [6]. Fertility restorer gene (*Rf*), was often found in these crop, can inhibit the expression of mitochondrial sterility gene. For the cotton, the gene are not consistent in different sterile lines.

The main cotton hybrids, which have the value of utilization were Harknessii cytoplasmic male sterile line, Trilobum cytoplasmic male sterile line, and cytoplasmic sterile line of upland cotton (104-7A, Xiangyuan A, Jin A). The three-line hybrid selection of China was primarily derived from the cytoplasmic male sterile lines of the upland cotton. Since the CMS sources are different, the restorers are also different, which leads to various theories on the CMS recovery mechanism of cotton. The fertility restoring characteristics of CMS in Harknessii cotton were regulated by one dominant gene, *Rf1*. The sterile nature of the Trilobum cotton could be restored by either *Rf2* of the Trilobum restorer gene or *Rf1* of the Harknessii restorer gene; however, *Rf2* is not able to restore the CMS-D2–2 of Harknessii. *Rf1* and *Rf2* are closely linked with a distance of 0.93 cM [7]. The Chinese breeding varies from the CMS lines of Harknessii and Trilobum. The fertility restoration of upland cotton CMS line is regulated by two pairs of independent recovery genes: *Rf1* completely dominant and *Rf2* partially dominant. The recovery effect of *Rf1* is higher than that of *Rf2* [8]. The identification of the molecular marker and gene mapping of CMS in cotton has also progressed. Liu et al. found 3 SSR and 2 RAPD markers closely linked to the restorer gene [9]. Feng et al. found that 3 STS was co-segregated from the restorer gene [10]. Yin et al. constructed accurate genetic and physical maps of 15 molecular markers closely linked to the restorer gene that was located on chromosome 19 (LGD08 linkage group) with a genetic distance of <1 cM, and the physical location was on 100 kb between the two BAC clone overlapping regions [11]. Wang et al. suggested that the two *Rf* restorer genes might be located on chromosome 19 in the D chromosome subgroup, i.e. chromosome D5 of the cotton [12, 13]. However, due to differences in the source of sterile cytoplasm and the variation in nuclear genotypes, the effects of nuclear gene and sterile cytoplasm are different. Thus, fine positioning and finding new restorer gene candidates in upland cotton are essential.

Large-scale genotyping plays a major role in genetic association studies. Specific locus amplified fragment sequencing (SLAF-seq) provides a high-resolution strategy for large-scale genotyping and can be applied to various species and populations [14]; for instance, cucumber [15], *Glycine max* [16], and sesame [17]. It is based on reduced representation library (RRL) and high-throughput sequencing. The technology has several distinguishing characteristics: i) deep sequencing to ensure genotyping accuracy; ii) reduced representation strategy to reduce sequencing costs; iii) pre-designed reduced representation scheme to optimize marker efficiency; and iv) double barcode system for large populations [14].

In this study, we used the female parent CMS line 3096A (using CMS 104-7A of upland cotton as a recurrent parent line that was breeded with the backbone parent line for nucleus replacement) of three-line Ji FRH3018 [18] and the male parent restorer line 866R with strong restoring power and its combination F_2_ segregating population as the material. Herein, we studied the fine mapping of the restorer gene and its correlated candidate gene using high-throughput sequencing platforms. A total of 137,741 SNPs were detected and we found that 20 candidate genes are identified and 19 genes were found annotated in each database of the candidate genes located on 1.35Mbp of chrD05.

## Methods

### Test material

The female parent CMS line 3096 from three-line CMS hybrid Ji FRH3018 of upland cotton and the male parent restorer line 866 with strong restoring power and its combination F_2_ segregation population (30 + 30 mixed pools with extreme characteristics) were used as research materials.

### Test method

#### Genomes resequencing of CMS line 3096A and fertility restorer line 866R

##### Sample collection and SLAF library preparation

Fresh leaves were obtained from the parent lines and F_2_, frozen with liquid nitrogen, extracted by the CTAB method, and assessed for the quality of DNA by 1% agarose gel electrophoresis. The purity of DNA was examined using the NanoPhotometer® spectrophotometer (Implen, CA, USA). The DNA concentration was estimated using Qubit® DNA Assay Kit in Qubit® 2.0 Fluorometer (Life Technologies, CA, USA).

We used 1.5 μg DNA/sample as input material for the preparations of the sample. We have chosen to use *RsaI*as restriction enzyme in the electronic enzyme-digestion projections to the reference genome sequences of cotton. Sequencing libraries were generated using *RsaI*of restriction enzyme according to the manufacturer’s recommendations, and index codes were added to ascribe the sequences to each sample. Briefly, the DNA sample was fragmented by sonication to a size of 350 bp. Then, the DNA fragments were end-polished, A-tailed, and ligated with the full-length adapter for Illumina sequencing by PCR amplification. Consequently, the PCR products were purified (Agencount® AMPure® XP, USA), and libraries were analyzed for size distribution by Agilent2100 Bioanalyzer and quantified by real-time PCR.

##### Illumina sequencing

The libraries constructed above were sequenced by Illumina HiSeq ™2500 (Illumina, Inc., San Diego, USA) platform at Biomarker Technologies Corporation in Beijing (http://www.biomarker.com.cn/) and 125 bp paired-end reads were generated with an insert size approximately 350 bp.

##### Data analysis, data filtering, and alignment

The recently released genome of *Gossypium hirsutum* was downloaded from Cotton Research Institute (CRI) of Nanjing Agricultural University in China. (http://mascotton.njau.edu.cn/Data.htm, v1.1) and used as a reference genome [19]. Fastx-toolkit (v 0.0.14–1) was used to filter out the low-quality reads based on the following criteria: (i) reads with ≥10% unidentified nucleotides (N); (ii) reads >50% read length with a Phred quality value ≤10; (iii) reads with the adapter. The remaining clean reads were aligned to the reference cabbage genome using BWA-MEM (0.7.10-r789) [20] and default parameters. Sequence Alignment/Map tools (SAMtools) (v1.1) [21] was applied to sort and index the resulting binary alignment map (BAM) format files. The duplicates were excluded using Picard tools (v1.102) (http://broadinstitute.github.io/picard/), and the final sorted bam files were utilized in the downstream analysis. Variant calling and filtering were performed in order to reduce the inaccuracy of the alignment. The local realignment around insertions and deletions, the base quality recalibration of the reads and variant calling was conducted using GATK Tools version 3.6. GATK Haplotype Caller (HC) was used for variant calling [22, 23]. The variants that fulfilled the following criteria were retained (1) mapping quality filter equivalent to PASS; (2) quality depth (QD) >2; (3) mapping quality (MQ) >40; (5) QUAL >30. Moreover, the variants were filtered further if the coverage was <10, the cluster SNPs were >2 in a 5 bp window, if the SNP around the Indel was within 5 bp. SV detection and annotation BreakDancer was used to predict the five types of structural variants (SVs): insertions (INSs), deletions (DELs), inversions (INVs), intra-chromosomal translocations (ITXs), and inter-chromosomal translocations (CTXs) from next-generation paired-end sequencing reads utilizing the read pairs mapped with excessive separation distances or orientation. The SVs with read depth < 2 were filtered. Bedtools was employed to annotate the detected DELs, INSs, and INVs. The detection and annotation of CNVs (copy number variations) refers to a normal variation in the number of copies of ≥1 sections of some genomic fragments. We used CNVnator (parameters: -call 100) for the identification of CNVs and bedtools for annotations.

#### SLAF library construction and high-throughput sequencing

The target fragment was selected by PCR amplification, purification, sample mixing, and excising from the gel. Illumina HiSeq™2500 was utilized for sequencing after inspection of the quality of the library.

#### SLAF tag development and SNP detection

The original data reads were obtained by dual-index sequencing for each sample. After filtering the sequencing joints of the reads, the sequencing quality, and the volume of data were assessed. The efficiency of *Rsa I* through the control data was used to determine the accuracy and efficiency of the test procedure. The data reads were compared to that of the reference genome and the SLAF tag was developed in parent lines and mixed pools in order to identify the SLAF tag with a polymorphism in parent lines and SNP with reads coverage [21]. A correlation analysis was conducted to identify the SNPs on the loci closely related to the characteristics and determine the candidate regions according to the correlation thresholds. Finally, a functional annotation and biological pathway enrichment analysis were conducted to identify the genes in the candidate regions.

#### Correlation analysis

##### SNP-index analysis

The SNP-index of the two mixed pools was calculated using the SNP data of the parent lines and assessing the loci that might be associated with the segregation of characteristics through the ΔSNP-index [24, 25]. The SNP-index is calculated as follows:

$$ {\displaystyle \begin{array}{l}\mathrm{SNP}\hbox{-} \mathrm{index}\ \left(\mathrm{Mut}\right)=\uprho \mathrm{x}/\left(\uprho \mathrm{X}+\uprho \mathrm{x}\right)\\ {}\mathrm{SNP}\hbox{-} \mathrm{index}\ \left(\mathrm{WT}\right)=\uprho \mathrm{x}/\left(\uprho \mathrm{X}+\uprho \mathrm{x}\right)\\ {}\Delta \mathrm{SNP}\hbox{-} \mathrm{index}=\mathrm{SNP}\hbox{-} \mathrm{index}\ \left(\mathrm{Mut}\right)\hbox{-} \mathrm{SNP}\hbox{-} \mathrm{index}\ \left(\mathrm{WT}\right)\end{array}} $$

Mut and WT are the mutation and wild-type pool of the filial generation, respectively. ρX and ρx indicate the number of reads of the alleles of the wild and the mutation parent lines appearing in their pools, respectively. The difference in each locus between the mutation and pools can be observed through the ΔSNP-index [26]. In order to eliminate the false positive locus, the SNP-indexes marked on the same chromosome can be fit by the position of the marker on the genome. The region above the threshold is correlated to the parameters. With respect to the qualitative character, the correlation threshold is the theoretical ΔSNP-index value of the corresponding population. For example, the correlation threshold of the F_2_ population is 0.67. In the case of quantitative character the correlation threshold is obtained by a computer simulation sampling experiment, and the probability of each marker associated with the target characteristic is calculated.

#### Euclidean distance (ED) algorithm

The ED algorithm evaluates the significant difference between mixed pools using the sequencing data. It also evaluates the area associated with the specific parameter [27]. Theoretically, in addition to the difference in the target character-related loci between the two mixed pools established by BSA, the others tend to be consistent, and hence, the ED value of the non-target related loci is equivalent to 0. The formula for ED is as follows:

$$ ED=\sqrt{{\left( Amut- Awt\right)}^2+{\left( Cmut- Cwt\right)}^2+{\left( Gmut- Gwt\right)}^2+{\left( Tmut- Twt\right)}^2} $$

The larger the ED value, the greater the difference between the two mixed pools. Amut is the frequency of the A base in the mutation pool, and Awt is the frequency of the A base in the wild pool; Cmut is the frequency of the C base in the mutation pool, Cwt is the frequency of the C base in the wild pool; Gmut is the frequency of the G base in the mutation pool, Gwt is the frequency of the G base in the wild pool; Tmut is the frequency of the T base in the mutation pool, Twt is the frequency of the T base in the wild pool.

In the analysis, the SNP loci with differences in the genotypes between the two mixed pools are used for calculating the depth of each base in the different pools and the ED value of each locus. The original ED value is processed such as to exclude the background interference. In order to eliminate the false positives, the position of the marker on the genome can be utilized to fit the labeled ED on the same chromosome and select the region above the threshold as the region related to the fertility restoring gene according to the association threshold. In order to eliminate the false positive locus, the ED values marked on the same chromosome can be fit according to the position of the marker on the genome. The region above the threshold is selected as the region related to the fertility restoring gene according to the correlation threshold.

### Identification of potential candidate genes

The reference genome sequence of the AD genome of tetraploid *G. hirsutum* was downloaded. The region related to the target characteristics was identified in both genome sequences and scanned for annotated genes using the Multiple Sequence Comparison by Log-Expectation software.

The Method of InDel (insertion-deletion Length Polymorphism) Markers Development on the Correlated Region.

Eprimer3 in the EMBOSS (v6.4.0) [28] software package was used on both ends of these loci sequences to design primers. The PCR reaction system constituted of 25 μL, containing 2 mmol/L MgCl2, 100 μmol/L dNTP, 0.2 μmol/L primers, 2 U Taq polymerase, 50 μL template DNA, and overlying 20 μL mineral oil. The PCR reaction was carried out in type PE480 DNA amplification equipment at 94 °C degeneration 3 min, 94 °C modified 30s, 40s, 58 °C annealing stretching up to 72 s, and 72 °C for 40 cycles, followed by a final extension at 72 °C for 10 min. The PCR products were resolved on 6% polyacrylamide electrophoresis.

## Results and analysis

### SLAF-seq data analysis and evaluation

The two parent lines and F_2_ segregation population were sequenced by SLAF-seq. *Rsa I* is selected to construct the SLAF library, and the SLAF fragment should be between 364 and 414 bp; 38.94 M reads were obtained. The reads from samples were aligned to the reference genome using the BWA software, with >80% efficiency, which is normal. For sequencing results, the average Q30 was 92.01%, and the average GC content was 37.63%. The male parent lines (R restorer lines) retrieved 9,673,045 reads, Q30 was 90.07%, and the average GC content was 37.40%. On the other hand, the female parent lines (A sterile lines) obtained 9,901,640 reads, Q30 90.65%, and the average GC content was 37.41%. The filial generation F_2_ (aa and ab) retrieved 10,687,924 and 8,679,918 reads, respectively, Q30 was 93.73% and 90.04%, respectively, and the average GC content was 37.96% and 37.73%, respectively (Table 1).

**Table 1:** Mining results of the high-throughput sequencing data

Sample ID	Total map (%)	Properly mapped (%)	Total Reads	Q30 percentage (%)	GC percentage (%)
R	99.11	95.42	9,673,045	90.07	37.4
A	99.35	95.77	9,901,640	90.65	37.41
aa	99.18	95.41	10,687,924	93.73	37.96
ab	99.41	95.76	8,679,918	90.04	37.73

### Development of SLAF tag and SNP

A total of 165,007 SLAF tags have been developed. The average sequencing depth of the parent lines was 47.90× and that of the mixed pools was 50.78×. Of these, the male parent lines obtained 16,173 SLAF tags with an average sequencing depth of 46.01×. The female parent lines obtained 161,854 SLAF tags, and the average sequencing depth was 49.78×; whereas, the filial generation F_2_ retrieved 163,688 and 163,189 SLAF tags, respectively, and the average sequencing depth was 55.96× + 45.59× (Table 2).

**Table 2:** Sequencing data of the developed SLAF markers

Sample ID	SLAF number	Total depth	Average depth
R	161,173	7,415,507	46.01×
A	161,854	8,057,541	49.78×
aa	163,688	9,159,461	55.96×
ab	163,189	7,440,285	45.59×

**Fig. 1:**
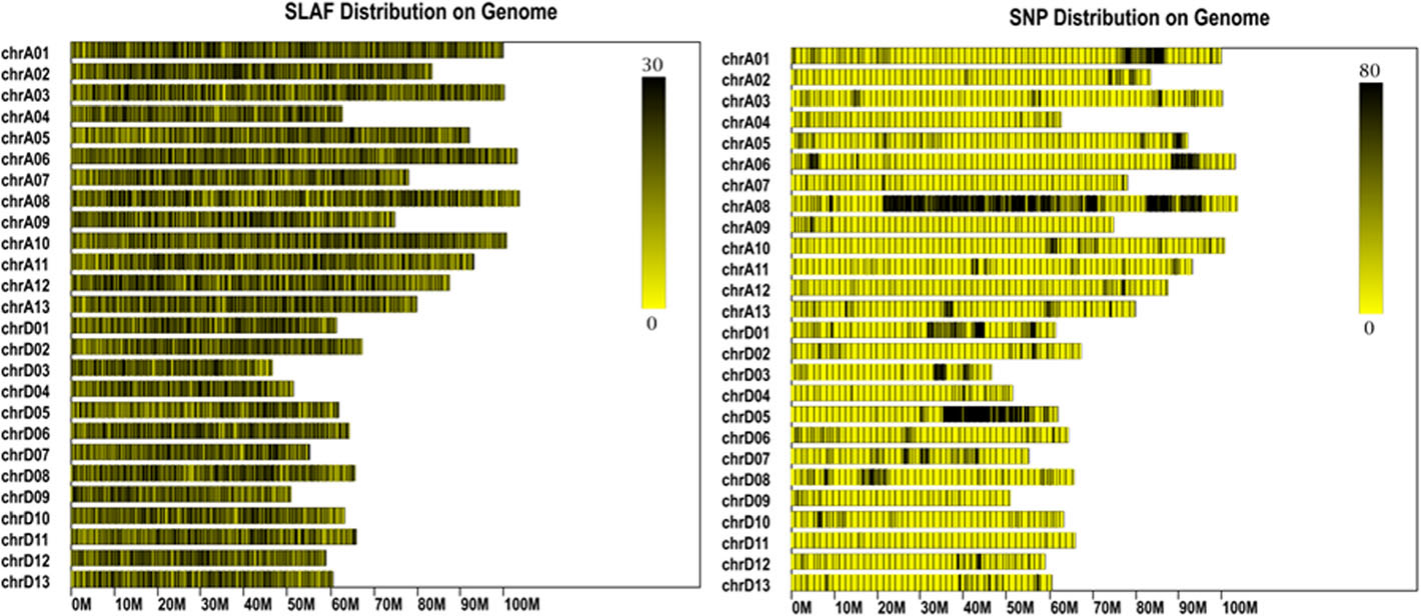
SLAF distribution and SNP markers on chromosome. Note: The abscissa is the length of the chromosome. Each yellow band represents a chromosome. The genome is divided by every 1Mbp. The more the number of SLAF tags in each window, the deeper the color and lesser the number of SLAF tags, the lighter the color. The darker area in the figure is the area where the SLAF tags are centrally distributed. The left panel shows the distribution of the SLAF tag, and the right panel is the distribution of SNP

SNPs were primarily detected by GATK software. According to the positioning results of the sequencing reads to the reference genome, GATK performs the local realignment, GATK mutation detection, samtools mutation detection, and identifying the overlapped mutation loci of GATK and samtools in order to ensure the accuracy of SNP, and obtain the final SNP loci set. A total of 137,741 SNPs were detected, of which, the male parent SNPs were 113,311, and the heterozygosity of SNPs in the sample was 4.19%. The female parent SNPs were 98, 861, and the heterozygosity was 5.37%. The filial generation F_2_ demonstrated 82,874 and 75,961 SNPs, respectively, and the heterozygosity was 20.55 and 19.28%, respectively (Table 3). The distribution of SLAF tags and SNP markers on different chromosomes was enumerated (Additional file 1), chrA01 had the maximum number of SLAF tags, while chrA08 exhibited the maximum number of SNP markers. According to the distribution of SLAF and SNP on the chromosome, the chromosome distribution map of SLAF tag and SNP is plotted. The specific distribution is shown in Fig. 1.

**Table 3:** The statistic results of each sample SNP

Sample ID	Total SNP	SNP number	Heterozygous locus numbers ratio (%)
R	137,741	113,311	4.19
A	137,741	98,861	5.37
aa	137,741	82,874	20.55
ab	137,741	75,961	19.28

Note: Total SNP: Total number of SNP is detected, SNP num: The number of SNPs in the corresponding samples detected, Heterozygous locus numbers ratio (%):The heterozygous locus numbers account for the proportion of all locus of SNPs in the sample

### Correlation analysis by SNP-index and ED

**Fig. 2:**
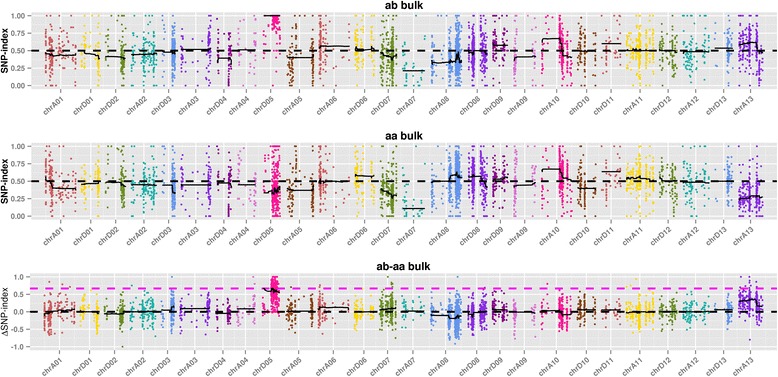
The distribution of SNP-index-associated values on chromosome. Note: The abscissa is the chromosome name. The color point represents the calculated SNP-index (or ΔSNP-index) value, and the black line is the fitted SNP-index (or ΔSNP-index) value. The top graph illustrates the distribution of the SNP-index values in h mixed pool; the middle graph is the distribution of the SNP-index values in L mixed pool; the bottom graph is the distribution of the ΔSNP-index values, where the magenta line represents the theoretical threshold line

Before the correlation analysis by SNP-index, 137,741 SNPs are filtered. A total of 16 SNP loci with multiple mutations are also filtered out. 102,105 loci with reads support <4 in the mixed pools are filtered out, and 27,289 loci that do not exist in the parent lines are filtered out. Finally, 8331 SNPs were obtained for the follow-up analysis. Using the SNP-index method, the correlation threshold was 0.67 according to the theoretical separation ratio of the experimental population. 20 association regions (Fig. 2) containing the genes were obtained, located at chr D05.

**Fig. 3:**
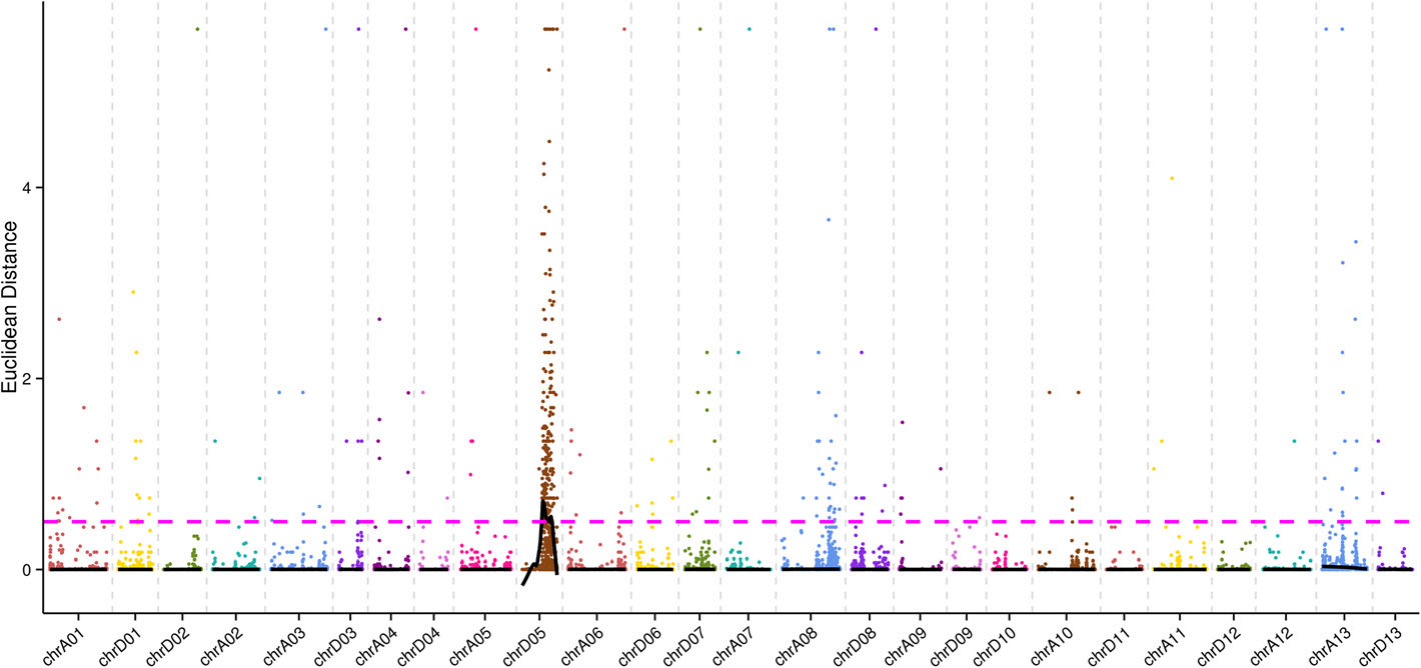
The distribution of ED-associated values on chromosome. Note: The abscissa is the chromosome name. The color point represents the ED value of each SNP locus. The black line is the fitted ED value, and the red dotted line represents the significantly associated threshold. The higher the ED value, the better the correlation effect

Similarly, before correlation analysis by ED, 137,741 SNPs should also be filtered out. 102,114 loci with read support <4 in any mixed pool are first filtered out, resulting in 35,627 high-quality and reliability loci. Therefore, a total of 14,226 different loci were identified between the two mixed pools. The correlation value was calculated by ED, and the median + 3SD of all the loci fitted values was considered as the correlation threshold of the analysis: 0.4969. A total of 351 correlated genes (Fig. 3) were obtained according to the correlation threshold.

Finally, the intersection of the associated genes obtained from the above two methods was found to be located on the candidate gene on 1.35 Mb of chrD05, and about 20 candidate genes were identified (Table 4). A correlation analysis of the genetic information to the associated region is summarized in the Additional file 2.

**Table 4:** The information of the association region

Assocition region	Chromosome ID	Start	End	Size (Mb)	Gene number
I	chrD05	37,535,705	37,755,211	0.22	6
II	chrD05	39,558,551	40,416,294	0.86	12
III	chrD05	40,531,406	40,804,095	0.27	2
	Total			1.35	20

### Gene functional annotations in related area correlation region

The 20 genes in the correlated region are compared to the databases of NR, SwissProt [29], GO [30], COG, and KEGG [31] using BLAST software. Finally, the annotations of 19 genes were obtained (Additional file 3). A total of 19/20 genes were found annotated in each database. Of these, annotations of 8 genes in KEGG, participating in 10 signaling pathways were found, including plant hormone signal transduction, protein output, DNA replication, homologous recombination, mismatch repair, nucleotide excision repair, ribosome, nitrogen metabolism, purine, and pyrimidine metabolism. In the DNA replication pathway, the enrichment factor 29.37 was a significant difference (*p* = 0.00186).

### Differences in sterile and restorer line on the correlated region

The genomes of the CMS line 3096A and fertility restorer line 866R were sequenced at 19× and 20× read depth, respectively, by Illumina sequencing of the paired-end libraries. Using the cotton AD-genome sequence as a reference, genetic variations, single base mutations, insertions, and deletions as compared to the reference genome were identified. The comparison of the structure of the genomes of the sterile and restorer lines on the correlated region revealed that the restorer line was located on the SV of the correlated region; however, the sterile line was not found on the SV as compared to the reference genome. We found that 7 SVs, 4 SVs are deletion and 3 SVs are interchromosomal translocation, are on the restorer line (Table 5). A total of 1607 indels were found in the correlated region, including 1246 intergenic indels, 3 exonic indels, involving 3 genes: *Gh_D05G3001*, *Gh_D05G3028*, and *Gh_D05G3039*; we found 242 intronic indels, 51 upstream indels and 65 downstream indels. A total of 13,175 SNP loci exhibited differences in the correlated region of the sterile and restorer lines, including 10,711 intergenic SNPs, 1858 intronic SNPs, 254 upstream SNPs, 227 downstream SNPs, 124 exonic SNPs and 2 splicing SNPs in reference to the genes, *Gh_D05G3005* and *Gh_D05G3038*. Nonsynonymous SNPs were found in 16 exonic regions, 1 stop-gain SNP was identified in *Gh_D05G3042*, and a stop-loss SNP was discovered in *Gh_D05G3031*.

**Table 5:** The SV on the correlated region in restorer lines

Chr1	Pos1	Orient-ation1	Chr2	Pos2	Orient-ation2	Type	Size	Score	num_Reads
D05	40000632	14 + 0-	D05	40005953	0 + 16-	DEL	5334	99	14
D05	40285089	15 + 0-	D05	40286963	1 + 17-	DEL	1931	99	15
D05	40356478	17 + 0-	D05	40356891	0 + 15-	DEL	473	99	13
D05	40643903	13 + 10-	D05	40655906	0 + 12-	DEL	12091	99	12
D05	39644894	7 + 15-	scaffold1082_A05	9062	0 + 14-	CTX	−318	99	12
D05	37580919	0 + 15-	scaffold4041_D05	13444	15 + 0-	CTX	−318	99	15
D05	39824830	14 + 0-	scaffold6268	11207	12 + 14-	CTX	−318	99	12

### InDel (insertion-deletion length polymorphism) markers development on the correlated region

**Fig. 4:**
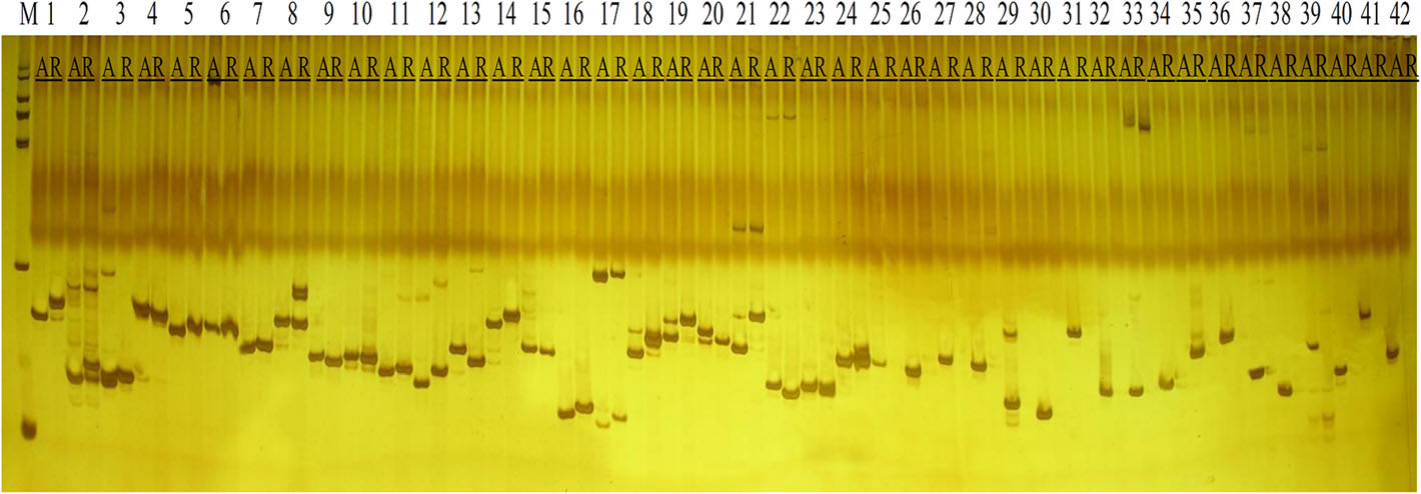
The polymorphic graph of primers. Note: 1–24 Codominant markers 25–42 Dominant markers A: sterile lines R: restorer line

The analysis of the comparison of the correlated regions on the sterile and restorer lines’ genome sequence found 1607 InDel sites. While analyzing the sterile and maintainer line amplification of the genomic DNA and design 165 primers, we found 42 primers (Attached Additional file 4) that distinctly detected the polymorphism, and hence, could be used as InDel markers. The 42 InDel markup tags, 24 as codominant markers, and 18 as dominant markers were developed Fig. 4. These will be laid as the underlying foundations for the fine mapping of the restorer genes.

## Discussion

### The molecular marker discovery and fine mapping of fertility restoring gene of CMS in cotton

The molecular marker discovery and fine mapping of fertility restorer gene of CMS in cotton are under intensive research. Yin et al. established the location of *Rf1* on 100 kb between two BAC clone overlapping regions and selected 5 SSR in proximity to *Rf1* by constructing a BAC library of *Gossypium harknessii* cytoplasmic male sterile restorer lines coupled with the genetic and physical maps recovering gene linkage [11]. Yang et al. screened out 6 EST-SSR markers (NAU2650, NAU2924, NAU3205, NAU3652, NAU3938, and NAU4040) with 0.327 cM from the fertility restorer *Rf1* of CMS in Harknessii cotton [32]. Wu et al. found that the fertility of CMS-D2 was regulated by a pair of dominant single gene *Rf1*, and 13 molecular markers closely linked to the fertility were screened out. The marker closest to *Rf1* was BNL3535 with a genetic distance of 0.049 cM; on the other side NAU3652 was the nearest marker with a genetic distance of 0.078 cM. [33]. Wang et al. demonstrated that CIR179–250 was closely linked to both *Rf1* and *Rf2*, which was located on LGD08 linkage group (D5 chromosome, 19th chromosome) of D genome set with CMS-D2 and CMS-D8 restorer, respectively, of upland cotton used as research material [13]. Li et al. located Jin-A cytoplasmic male sterile restorer gene *Rf* on the 19th chromosome (LGD08) with a distance of 5.4 and 10.3 cM from markers CM042 and CIR179, respectively [34]. You et al. studied three cotton cytoplasmic male sterile lines and their corresponding restorers from China, Israel, and the USA, respectively. The results indicated that 2 restoring genes in the restorers were from the USA. The *Rf1* was positioned between BNL3535 and CIR179 at a distance of 5.3 cM, while *Rf2* was between STS659 and BNL1045 at a distance of 4.8 cM. Only 1 restoring gene was identified in the restorers from China, and *Rf* was between CIR222 and BNL632 at a distance of 6.7 cM. Only 1 restoring gene was found in the restorers from Israel, and *Rf* was between STS147 and CIR179 at a distance of 4.3 cM [35]. According to the SSR primers, we found the recovery of SSR markers in the gene location map (Table 6). Furthermore, we established that although the sterile line source type was different, the tags on the reference genome was found on chrD05 between 35,690,656–59,566,733. The present study on the fertility restoration gene identified the location for chr D05 base sequence as 37,535,705–37,755,211 (0.22Mbp), 39,558,551–40,416,294 (0.86Mbp), and 40,531,406–40,804,095 (0.27Mbp) interval; the sterility-related gene mapping was reported between NAU2924 and NAU4040 SSR markers. As the same markers appear in the position of cotton CMS fertility restoring gene from different sources, it is speculated that the chromosomal segments of the restoring gene derived from various types of restorer lines should be consistent. These markers, which are closely linked to the restorer gene, act as insertion or deletion of the restorer gene fragment in the process of genetic improvement, resulting in the altered genetic distance. The present study developed 42 InDel markers in the correlated region; subsequently, it should be the laid a foundation for positioning of the cotton fertility restoring genes. The present results also showed that SLAF-seq technology is an efficient and high-resolution QTL fine-positioning technique characterized by high success rate, specificity, stability, and cost-efficiency. The combination of SLAF-seq technology, SNP_index, and BSA provides an efficient method for identifying the genomic regions associated with the characteristics described above.

**Table 6:** The summary of restorer gene marker in the genome location

Marker	Chromosome ID	Genome location	Source	Restorer gene	Reference
NAU2924	D5	35690459–35690656	*Gossypium harknessii*	*Rf1*	Yang [32]
NAU3652	D5	37123844–37124070	*Gossypium harknessii*	*Rf1*	Yang [32] Wu [33]
NAU4040	D5	43363683–43363832	*Gossypium harknessii*	*Rf1*	Yang [32]
NAU2650	D5	44346401–44346571	*Gossypium harknessii*	*Rf1*	Yang [32]
NAU3205	D5	50573886–50573694	*Gossypium harknessii*	*Rf1*	Yang [32]
NAU3938	D5	52546928–52547146	*Gossypium harknessii*	*Rf1*	Yang [32]
BNL3535	D5	54287875–54288016	CMS-D2	*Rf1*	Wu [33]; You [35]
CIR222	D5	54288233–54287945	Unknown (China)	*Rf*	You [35]
CM042	D5	55139471–55139336	Jin A	*Rf*	Li [34]
BNL632	D5	59566733–59566464	Unknown (China)	*Rf*	You [35]

### Cloning of fertility restorer gene

The cloning of fertility restorer gene in cotton CMS is yet under investigation. Yang et al. identified the gene containing *Rf1* and conducted the whole length sequencing. The *Rf1* locus is found to contain 5 *PPR* genes and 2 genes highly homologous to the *PPR* gene in a region of approximately 130 kb. Based on gene prediction, characterization analysis, and the difference in the phylogenetic sequence analysis, ORF3 is speculated as the *Rf1* gene that encodes the *PPR* gene and contains the mitochondrial localization signal. ORF3 necessitates functional complementation by transgenesis [36]. Zhang et al. concluded that the starch synthase and the phosphate -ribose o-aminobenzoic acid transferase (*PAT*) gene might be associated with the *Rf2* gene in the Trilobum cytoplasm by differential display technique analysis [37]. Wu and Hou cloned the genes, *GH*182*Rorf*392 and *GhPG2*, related to cotton fertility restoration from the upland cotton restorer Y18R line. *GH*182*Rorf*392 encodes 392 amino acids. The 3′ end of the gene contains a 26 s rRNA sequence, and the 5′ end is a novel sequence [38, 39]. The gene might interact with ribosomes in organelles such as mitochondria or chloroplasts. *GhPG2* codes for polygalacturonase, which is related to the flower organ development. In recent years, the *Rf* genes of crops such as corn [40], rice [41], onion [42], and sorghum [43] have been cloned successively. Except for corn *Rf2* and *Rf4* and rice *Rf2* and *Rf17*, the other known *Rf* genes belong to the *PPR* (pentatricopeptide repeats) gene family. The coding protein of the *PPR* gene family is considered to be a single-stranded RNA-binding protein and plays a vital role in the processing of organelles’ RNA [44]. The *Rf* gene encoding protein plays a major role in organelle RNA processing. The N ends of the *Rf* gene encoding protein contains the mitochondrial localization sequences that are transported to mitochondria after maturing in the cytoplasm, participating in mitochondrial gene transcription, post-transcriptional processing, and translation for regulating the plant fertility [45]. These studies provided further references for exploring the cotton CMS fertility restorer genes.

In this study, 20 genes were screened out from the correlation region of the fertility restorer genome, including 1 gene with unknown function. Although the functional annotation and analysis of these candidate genes did not identify the *PPR* gene family, the gene of *Gh_D05G3001* encoding the trihelix transcription factor GT-1-like protein with myb-like protein domain was found, such that the myb-like protein play a major role in normal anther and pollen development [46]. In addition, *Gh_D05G3003* coding FAD-binding Berberine family protein was identified, and both *Gh_D05G3037* encoding the protein and 23 kDa jasmonate-induced protein-like protein in *Corchorus olitorius* are homologous. The *Gh_D05G3039* encoding the protein belongs to B-box and Zinc finger family protein; the above four genes are associated with tapetal development. The tapetum plays a crucial role in anther development by providing the essential enzymes and nutrients for pollen development. The tapetum, which is the innermost of the four sporophytic layers in the anther wall, comes in direct contact with the developing male gametophyte regulating the development and maturation of microspores [47, 48]. Wang et al. discovered that the abortion of 104-7A and Xiangyuan 4-A at the stage of meiosis, the abortive tapetum, sporogenous cells, and microspore mother cells were considered as chromosomal aberrations [49]. The research region of the annotation to the four tapetum development-related genes, especially with myb-like protein domain of *Gh_D05G3001* serves as a focal point for the next phase of research. Its function was similar to AtMYB103 and required for tapetal development and microsporogenesis, found in *Arabidopsis thaliana* [50].

In addition, the gene *Gh_D05G3036* encoding carbonic anhydrase 2-like may also play a pivotal role in the process of anther development [51]. The gene of *Gh_D05G3030* encoding the xyloglucan endotransglucosylase might be involved in the growth of stamen filaments [52]. The present study also found that both *Gh_D05G3042* and *Gh_D05G3043* are a series of homologous gene loci, that encode lipid phosphate phosphatase 2, which is a part of ABA signaling. The gene of *Gh_D05G3043* also harbors the mitochondrial sequence. Only a few studies have yet reported the role of these genes in the anther development. Thus, we aspire to substantiate their functional role in future studies.

### Standard criteria for restorer genes

Analyzing the characteristic controlled by a single gene or polygene according to the Mendelian classical genetics and molecular genetics might be challenging. When a series of genes that control a characteristic is clustered in a specific segment of the chromosome, the classical genetics might presume that the segment is one gene; however, the molecular genetics would divide the segment into several genes according to ORF, which increases the cloning difficulty of genes modulating these characteristics. In this study, we found that several genes in the associated region are related to the development of cotton anthers, and the restorer genes are difficult to be identified. The following questions are yet to be addressed in order to determine the restorer genes: (1) Why the restoring gene of the restorer lines can restore the infertility of sterile lines? (2) Why does the homologous gene of the restorer gene from maintainer line cannot restore the infertility of sterile lines? (3) The difference in the characteristics is caused by functional gene expression arises from the variability in the sequence of the upstream regulatory region; how is it determined as a restorer gene? Therefore, the study of restorer genes necessitates further analysis to understand the mechanism underlying the sterility of nuclear and cytoplasm interaction.

CMS is a common feature encountered in plant species, which is the result of a genomic conflict between the mitochondrial and nuclear genomes. CMS is caused by mitochondrial-encoded factors that can be counteracted by nuclear-encoded factors restoring male fertility [53]. Despite extensive research, the molecular mechanisms underlying male sterility are yet unknown, especially in the cotton. Li et al. discovered the molecule, *orf160*, unique at the downstream of *atp4* in the cytoplasm of the male sterile cotton lines (*Gossypium harknessii* L.). The full length of the gene was 480 bp, and the sequence at the N-terminal was partially homologous to the *atp6* sequence and that at the C-terminal was homologous to the nuclear sequence [54]. Suzuki et al. compared the RNA editing events of 8 genes (*atp1*, *atp4*, *atp6*, *atp8*, *atp9*, *cox1*, *cox2*, *cox3*) in the mitochondria from sterile lines, maintainer lines, and restorer lines and found that the relationship between sterility and fertility restoration cannot be explained by RNA editing analysis of these genes [55]. With the completion of the sequencing of the whole genome of the upland cotton [19, 56] and the cotton mitochondrial genome [57], the studies on cotton CMS and the fertility restoration mechanism can provide the information on the crosstalk between gene functions and genes of upland cotton at the global level.

Whether CMS fertility restoration is caused by gene mutation or gene regulation is yet to be substantiated.

## Conclusions

In this study, associated markers identified by super-BSA could accelerate the study of CMS in cotton, as well as in the other crops. Some of the 20 genes’ preliminary characteristics provided useful information for further studies on CMS crops.

## Additional files

Additional file 1: The summary of the SLAF tag and SNP marker on chromosomes. (DOC 41 kb)

Additional file 2: The information of genetic analysis onto the association region. (DOC 38 kb)

Additional file 3: The annotation of COG, KEGG, GO, Swiss-Prot and nr to the candidate genes. (DOC 51 kb)

Additional file 4: List of InDel marker primers. (DOC 51 kb)

## Abbreviations

BSA: Bulked segregantion analysis
CMS: Cytoplasmic male sterility
ED: Euclidean distance
PPR: Pentatricopeptide repeats
SLAF-seq: Specific length amplified fragment sequencing
SNP: Single nucleotide polymorphism
