# Supplementary material for: Physical mapping and candidate gene prediction of fertility restorer gene of cytoplasmic male sterility in cotton
Source: BMC Genomics. 2018 Jan 2;19:6. doi: 10.1186/s12864-017-4406-y (PMC5751606; doi:10.1186/s12864-017-4406-y)
Supplement: Supplementary file 1 — The summary of the SLAF tag and SNP marker on chromosomes. (DOC 41 kb) [file 12864_2017_4406_MOESM1_ESM.doc]

Additional file 1

The summary of the SLAF tag and SNP marker on chromosomes

| Chromosome ID | SLAF number | SNP number |
| --- | --- | --- |
| chrA01 | 6,990 | 6375 |
| chrA02 | 6033 | 2958 |
| chrA03 | 6806 | 3898 |
| chrA04 | 4301 | 2296 |
| chrA05 | 5900 | 4374 |
| chrA06 | 7025 | 5215 |
| chrA07 | 5223 | 2291 |
| chrA08 | 6934 | 20635 |
| chrA09 | 5090 | 2328 |
| chrA10 | 6910 | 3607 |
| chrA11 | 6157 | 3317 |
| chrA12 | 5945 | 3107 |
| chrA13 | 5492 | 3431 |
| chrD01 | 4299 | 4955 |
| chrD02 | 4669 | 2268 |
| chrD03 | 3204 | 2392 |
| chrD04 | 3503 | 1522 |
| chrD05 | 3766 | 10987 |
| chrD06 | 4520 | 2567 |
| chrD07 | 3735 | 3423 |
| chrD08 | 4556 | 3417 |
| chrD09 | 3367 | 1512 |
| chrD10 | 4240 | 2205 |
| chrD11 | 4225 | 1311 |
| chrD12 | 4035 | 2226 |
| chrD13 | 3959 | 2095 |
| Other | 34123 | 33029 |
| Total | 165007 | 137741 |
