# Supplementary material for: Physical mapping and candidate gene prediction of fertility restorer gene of cytoplasmic male sterility in cotton
Source: BMC Genomics. 2018 Jan 2;19:6. doi: 10.1186/s12864-017-4406-y (PMC5751606; doi:10.1186/s12864-017-4406-y)
Supplement: Supplementary file 2 — The information of genetic analysis onto the association region. (DOC 38 kb) [file 12864_2017_4406_MOESM2_ESM.doc]

Additional file 2

The information of genetic analysis onto the association region

| Association region | Chromo-some | Start sequence | End sequence | Gene ID |
| --- | --- | --- | --- | --- |
| I | D05 | 37582777 | 37588788 | ID=Gh_D05G3000; Name=Gh_D05G3000 |
|  | D05 | 37612036 | 37616050 | ID=Gh_D05G3001; Name=Gh_D05G3001 |
|  | D05 | 37629819 | 37630118 | ID=Gh_D05G3002; Name=Gh_D05G3002 |
|  | D05 | 37632969 | 37633181 | ID=Gh_D05G3003; Name=Gh_D05G3003 |
|  | D05 | 37644137 | 37648147 | ID=Gh_D05G3004; Name=Gh_D05G3004 |
|  | D05 | 37660193 | 37742098 | ID=Gh_D05G3005; Name=Gh_D05G3005 |
| II | D05 | 39618869 | 39619621 | ID=Gh_D05G3028; Name=Gh_D05G3028 |
|  | D05 | 39655398 | 39660410 | ID=Gh_D05G3029; Name=Gh_D05G3029 |
|  | D05 | 39727597 | 39728660 | ID=Gh_D05G3030; Name=Gh_D05G3030 |
|  | D05 | 39768243 | 39769822 | ID=Gh_D05G3031; Name=Gh_D05G3031 |
|  | D05 | 39769871 | 39770140 | ID=Gh_D05G3032; Name=Gh_D05G3032 |
|  | D05 | 39805077 | 39806032 | ID=Gh_D05G3033; Name=Gh_D05G3033 |
|  | D05 | 39806707 | 39808835 | ID=Gh_D05G3034; Name=Gh_D05G3034 |
|  | D05 | 40011383 | 40011655 | ID=Gh_D05G3035; Name=Gh_D05G3035 |
|  | D05 | 40143352 | 40148974 | ID=Gh_D05G3036; Name=Gh_D05G3036 |
|  | D05 | 40190863 | 40191908 | ID=Gh_D05G3037; Name=Gh_D05G3037 |
|  | D05 | 40376229 | 40379144 | ID=Gh_D05G3038; Name=Gh_D05G3038 |
|  | D05 | 40414267 | 40415957 | ID=Gh_D05G3039; Name=Gh_D05G3039 |
| III | D05 | 40531787 | 40536975 | ID=Gh_D05G3042; Name=Gh_D05G3042 |
|  | D05 | 40629589 | 40632357 | ID=Gh_D05G3043; Name=Gh_D05G3043 |
