# Supplementary material for: Physical mapping and candidate gene prediction of fertility restorer gene of cytoplasmic male sterility in cotton
Source: BMC Genomics. 2018 Jan 2;19:6. doi: 10.1186/s12864-017-4406-y (PMC5751606; doi:10.1186/s12864-017-4406-y)
Supplement: Supplementary file 3 — The annotation of COG, KEGG, GO, Swiss-Prot and nr to the candidate genes. (DOC 51 kb) [file 12864_2017_4406_MOESM3_ESM.doc]

Additional file 3

The annotation of COG, KEGG, GO, Swiss-Prot and nr to these candidate gene

| Gene_ID | COG_class_annotation | KEGG_annotation | GO_annotation | Swiss-Prot_annotation | nr_annotation |
| --- | --- | --- | --- | --- | --- |
| Gh_D05G3000 | Replication, recombination, and repair | K02321|0.0|rcu:RCOM_1081910|alpha DNA polymerase, putative | Molecular Function: DNA binding (GO:0003677); Molecular Function: DNA-directed DNA polymerase activity (GO:0003887); Cellular Component: nucleus (GO:0005634); Biological Process: DNA-dependent DNA replication (GO:0006261); Biological Process: regulation of gene expression (GO:0010468); Biological Process: histone lysine methylation (GO:0034968); Biological Process: regulation of cell cycle (GO:0051726) | -- | DNA polymerase alpha subunit B [*Gossypium arboreum*] |
| Gh_D05G3001 |  |  | Molecular Function: chromatin binding (GO:0003682); Molecular Function: sequence-specific DNA binding transcription factor activity (GO:0003700); Cellular Component: nucleus (GO:0005634); Biological Process: regulation of transcription, DNA-templated (GO:0006355); Biological Process: membrane fusion (GO:0006944); Molecular Function: identical protein binding (GO:0042802); Molecular Function: sequence-specific DNA binding (GO:0043565); Biological Process: Golgi vesicle transport (GO:0048193) | Trihelix transcription factor GT-1 GN=GT-1 OS=Arabidopsis thaliana (Mouse-ear cress) PE=1 SV=1 | Homeodomain-like superfamily protein [*Theobroma cacao*] |
| Gh_D05G3002 |  |  |  |  |  |
| Gh_D05G3003 |  |  | Molecular Function: electron carrier activity (GO:0009055); Molecular Function: oxidoreductase activity, acting on CH-OH group of donors (GO:0016614) | Reticuline oxidase-like protein (Precursor) GN=At4g20830 OS=Arabidopsis thaliana (Mouse-ear cress) PE=1 SV=2 | FAD-binding Berberine family protein [*Theobroma cacao*] |
| Gh_D05G3004 | -- | K03108|0.0|vvi:100244217|hypothetical protein LOC100244217 | Cellular Component: signal recognition particle, endoplasmic reticulum targeting (GO:0005786); Biological Process: SRP-dependent cotranslational protein targeting to membrane (GO:0006614); Molecular Function: 7S RNA binding (GO:0008312) | -- | Signal recognition particle 72 kDa [*Gossypium arboreum*] |
| Gh_D05G3005 | General function prediction only | K07052|1e-119|vvi:100252714|hypothetical protein LOC100252714 |  | -- | Putative yyaK [*Gossypium arboreum*] |
| Gh_D05G3028 |  |  | Cellular Component: membrane (GO:0016020) | F-box/kelch-repeat protein At1g23390 GN=At1g23390 OS=Arabidopsis thaliana (Mouse-ear cress) PE=2 SV=1 | F-box/kelch protein, putative [Theobroma cacao] |
| Gh_D05G3029 |  |  |  | -- | Hypothetical protein F383_04284 [*Gossypium arboreum*] |
| Gh_D05G3030 | -- | K14504|0.0|rcu:RCOM_1630040|hypothetical protein | Molecular Function: hydrolase activity, hydrolyzing O-glycosyl compounds (GO:0004553); Cellular Component: cell wall (GO:0005618); Cellular Component: Golgi apparatus (GO:0005794); Biological Process: cellular glucan metabolic process (GO:0006073); Biological Process: response to heat (GO:0009408); Biological Process: response to cold (GO:0009409); Cellular Component: plant-type cell wall (GO:0009505); Cellular Component: chloroplast (GO:0009507); Biological Process: response to wounding (GO:0009611); Biological Process: response to mechanical stimulus (GO:0009612); Biological Process: plant-type cell wall organization (GO:0009664); Biological Process: response to auxin (GO:0009733); Biological Process: response to brassinosteroid (GO:0009741); Biological Process: xylem development (GO:0010089); Biological Process: response to chitin (GO:0010200); Biological Process: xyloglucan metabolic process (GO:0010411); Molecular Function: xyloglucan:xyloglucosyl transferase activity (GO:0016762); Molecular Function: xyloglucan-specific endo-beta-1,4-glucanase activity (GO:0033946); Biological Process: cell wall modification (GO:0042545); Cellular Component: apoplast (GO:0048046);; Biological Process: root hair cell differentiation (GO:0048765); Biological Process: primary root development (GO:0080022); Molecular Function: xyloglucan endotransglucosylase activity (GO:0080039) | Xyloglucan endotransglucosylase/hydrolase protein 22 (Precursor) GN=MUA2.13 OS=Arabidopsis thaliana (Mouse-ear cress) PE=1 SV=1 | Xyloglucan endotransglucosylase/hydrolase 22 -like protein [*Gossypium arboreum*] |
| Gh_D05G3031 | General function prediction only | -- | Biological Process: microtubule cytoskeleton organization (GO:0000226); Molecular Function: DNA binding (GO:0003677); Molecular Function: sequence-specific DNA binding transcription factor activity (GO:0003700); Molecular Function: GTPase activity (GO:0003924); Molecular Function: transporter activity (GO:0005215); Molecular Function: copper ion binding (GO:0005507); Molecular Function: protein binding (GO:0005515); Molecular Function: GTP binding (GO:0005525); Cellular Component: nucleus (GO:0005634); Cellular Component: mitochondrion (GO:0005739); Cellular Component: vacuole (GO:0005773); Cellular Component: Golgi apparatus (GO:0005794); Cellular Component: cytosol (GO:0005829); Cellular Component: plasma membrane (GO:0005886); Biological Process: glucose catabolic process (GO:0006007); Biological Process: DNA methylation (GO:0006306); Biological Process: methylation-dependent chromatin silencing (GO:0006346); Biological Process: regulation of transcription, DNA-templated (GO:0006355); Biological Process: protein ADP-ribosylation (GO:0006471); Biological Process: N-terminal protein myristoylation (GO:0006499); Biological Process: intracellular protein transport (GO:0006886); Biological Process: response to osmotic stress (GO:0006970); Biological Process: tubulin complex assembly (GO:0007021); Biological Process: small GTPase mediated signal transduction (GO:0007264); Biological Process: cell-cell signaling (GO:0007267); Biological Process: embryo sac cellularization (GO:0009558); Biological Process: virus induced gene silencing (GO:0009616); Biological Process: gravitropism (GO:0009630); Biological Process: response to auxin (GO:0009733); Biological Process: auxin-activated signaling pathway (GO:0009734); Biological Process: embryo development ending in seed dormancy (GO:0009793); Biological Process: endosperm development (GO:0009960); Biological Process: leaf senescence (GO:0010150); Biological Process: production of ta-siRNAs involved in RNA interference (GO:0010267); Biological Process: protein transport (GO:0015031); Molecular Function: phospholipase activator activity (GO:0016004); Biological Process: vesicle-mediated transport (GO:0016192); Molecular Function: hydrolase activity (GO:0016787); Biological Process: production of miRNAs involved in gene silencing by miRNA (GO:0035196); Biological Process: single-organism transport (GO:0044765); Biological Process: response to cadmium ion (GO:0046686); Molecular Function: protein dimerization activity (GO:0046983) | ADP-ribosylation factor GN=ARF1 OS=Zea mays (Maize) PE=2 SV=2 | PREDICTED: ADP-ribosylation factor 2-like [*Elaeis guineensis*] |
| Gh_D05G3032 | General function prediction only | K07977|1e-28|zma:100282739|ADP-ribosylation factor | Molecular Function: DNA binding (GO:0003677); Molecular Function: sequence-specific DNA binding transcription factor activity (GO:0003700); Molecular Function: transporter activity (GO:0005215); Molecular Function: copper ion binding (GO:0005507); Molecular Function: protein binding (GO:0005515); Molecular Function: GTP binding (GO:0005525); Cellular Component: intracellular (GO:0005622); Cellular Component: nucleus (GO:0005634); Cellular Component: vacuole (GO:0005773); Cellular Component: Golgi apparatus (GO:0005794); Cellular Component: cytosol (GO:0005829); Cellular Component: plasma membrane (GO:0005886); Biological Process: glucose catabolic process (GO:0006007); Biological Process: DNA methylation (GO:0006306); Biological Process: methylation-dependent chromatin silencing (GO:0006346); Biological Process: protein ADP-ribosylation (GO:0006471); Biological Process: N-terminal protein myristoylation (GO:0006499); Biological Process: intracellular protein transport (GO:0006886); Biological Process: small GTPase mediated signal transduction (GO:0007264); Biological Process: cell-cell signaling (GO:0007267); Biological Process: embryo sac cellularization (GO:0009558); Biological Process: virus induced gene silencing (GO:0009616); Biological Process: response to auxin (GO:0009733); Biological Process: embryo development ending in seed dormancy (GO:0009793); Biological Process: endosperm development (GO:0009960); Biological Process: leaf senescence (GO:0010150); Biological Process: production of ta-siRNAs involved in RNA interference (GO:0010267); Molecular Function: phospholipase activator activity (GO:0016004); Biological Process: vesicle-mediated transport (GO:0016192); Biological Process: production of miRNAs involved in gene silencing by miRNA (GO:0035196); Biological Process: response to cadmium ion (GO:0046686) | ADP-ribosylation factor 2 GN=OJ1119_H02.17 OS=Oryza sativa subsp. japonica (Rice) PE=2 SV=2 | PREDICTED: ADP-ribosylation factor 2-like isoform X1 [*Solanum tuberosum*] |
| Gh_D05G3033 |  |  |  | Uncharacterized protein At2g29880 GN=At2g29880 OS=Arabidopsis thaliana (Mouse-ear cress) PE=2 SV=1 | hypothetical protein F383_20963 [Gossypium arboreum] |
| Gh_D05G3034 |  |  |  | -- | Trimethylamine-N-oxide reductase [Gossypium arboreum] |
| Gh_D05G3035 | Translation, ribosomal structure and biogenesis | K02956|1e-46|pop:POPTR_935635|hypothetical protein | Molecular Function: structural constituent of ribosome (GO:0003735);; Cellular Component: ribosome (GO:0005840);; Biological Process: translation (GO:0006412);; Cellular Component: chloroplast (GO:0009507);; | 30S ribosomal protein S15, chloroplastic GN=rps15 OS=Gossypium hirsutum (Upland cotton) PE=3 SV=1 | ribosomal protein S15 (chloroplast) [Gossypium raimondii] |
| Gh_D05G3036 | Inorganic ion transport and metabolism | K01673|1e-143|rcu:RCOM_0528440|carbonic anhydrase, putative (EC:4.2.1.1) | Molecular Function: carbonate dehydratase activity (GO:0004089);; Cellular Component: mitochondrion (GO:0005739);; Cellular Component: cytosol (GO:0005829);; Cellular Component: plasma membrane (GO:0005886);; Molecular Function: zinc ion binding (GO:0008270);; Cellular Component: chloroplast stroma (GO:0009570);; Cellular Component: chloroplast envelope (GO:0009941);; Biological Process: response to carbon dioxide (GO:0010037);; Biological Process: regulation of stomatal movement (GO:0010119);; Biological Process: response to nitrate (GO:0010167);; Biological Process: nitrate transport (GO:0015706);; Biological Process: carbon utilization (GO:0015976);; Biological Process: methylglyoxal catabolic process to D-lactate (GO:0019243);; | Carbonic anhydrase, chloroplastic (Precursor) OS=Nicotiana tabacum (Common tobacco) PE=2 SV=1 | Carbonic anhydrase, chloroplastic [Gossypium arboreum] |
| Gh_D05G3037 |  |  |  | -- | uncharacterized protein LOC107903741 isoform X1 [Gossypium hirsutum],  23 kDa jasmonate-induced protein-like protein [Corchorus olitorius] |
| Gh_D05G3038 | Replication, recombination and repair | K07466|6e-81|rcu:RCOM_0528470|hypothetical protein | Cellular Component: cytosol (GO:0005829);; Biological Process: ubiquitin-dependent protein catabolic process (GO:0006511);; Biological Process: photorespiration (GO:0009853);; Biological Process: response to misfolded protein (GO:0051788);; Biological Process: proteasome core complex assembly (GO:0080129);; | Uncharacterized protein At4g28440 GN=At4g28440 OS=Arabidopsis thaliana (Mouse-ear cress) PE=1 SV=1 | hypothetical protein F383_14249 [Gossypium arboreum] |
| Gh_D05G3039 | General function prediction only | -- | Molecular Function: nucleic acid binding (GO:0003676);; Biological Process: regulation of transcription, DNA-templated (GO:0006355);; | Zinc finger CCCH domain-containing protein 15 GN=At1g68200 OS=Arabidopsis thaliana (Mouse-ear cress) PE=2 SV=1 | Zinc finger C-x8-C-x5-C-x3-H type family protein, putative isoform 3 [Theobroma cacao] |
| Gh_D05G3042 | Lipid transport and metabolism | -- | Molecular Function: acid phosphatase activity (GO:0003993);; Cellular Component: cytoplasm (GO:0005737);; Cellular Component: integral component of plasma membrane (GO:0005887);; Biological Process: phospholipid metabolic process (GO:0006644);; Biological Process: metabolic process (GO:0008152);; Molecular Function: phosphatidate phosphatase activity (GO:0008195);; Biological Process: abscisic acid-activated signaling pathway (GO:0009738);; | Lipid phosphate phosphatase 2 GN=F9L1.2 OS=Arabidopsis thaliana (Mouse-ear cress) PE=2 SV=1 | Lipid phosphate phosphatase 2 -like protein [Gossypium arboreum] |
| Gh_D05G3043 | Lipid transport and metabolism | -- | Molecular Function: acid phosphatase activity (GO:0003993);; Cellular Component: cytoplasm (GO:0005737);; Cellular Component: integral component of plasma membrane (GO:0005887);; Biological Process: phospholipid metabolic process (GO:0006644);; Molecular Function: phosphatidate phosphatase activity (GO:0008195);; Biological Process: abscisic acid-activated signaling pathway (GO:0009738);; Cellular Component: membrane (GO:0016020);; | Putative lipid phosphate phosphatase 3, chloroplastic (Precursor) GN=LPP3 OS=Arabidopsis thaliana (Mouse-ear cress) PE=2 SV=1 | Putative lipid phosphate phosphatase 3, chloroplastic -like protein [Gossypium arboreum] |
