# Supplementary material for: Physical mapping and candidate gene prediction of fertility restorer gene of cytoplasmic male sterility in cotton
Source: BMC Genomics. 2018 Jan 2;19:6. doi: 10.1186/s12864-017-4406-y (PMC5751606; doi:10.1186/s12864-017-4406-y)
Supplement: Supplementary file 4 — List of InDel marker primers. (DOC 51 kb) [file 12864_2017_4406_MOESM4_ESM.doc]

Additional file 4

List of InDel marker primers

| Number | PRIMER_ID | PRIMER_LEFT_SEQUENCE | PRIMER_RIGHT_SEQUENCE |
| --- | --- | --- | --- |
| 1 | D05_40311294_2 | GTGGTCGGTTATTATAGGCT | ACATGCAGTAGACGATCAAG |
| 2 | D05_37624830_5 | TAGTGGTATCAGAGCTAGGG | TTCTATCTCACTCGACTCGA |
| 3 | D05_37645201_2 | CCCTAGCCTGCAGAATTTTA | GTGTTGGGATTTTGCACTTT |
| 4 | D05_37741581_2 | AGTTGAGTACGGATAGGTGA | CATTGTGCTCGGAAAACATA |
| 5 | D05_39601065_4 | AATAACGTTCGCCCTATGAA | GACAATTCACCAATCCGTTC |
| 6 | D05_39607495_2 | CAGGTGTTGTCAGAAGGATT | GTAGCTCCACTAGTAATGCC |
| 7 | D05_39612311_2 | AAGATTGAATCGCATGGAGA | GCGAGATTTTCAGTTCCTTG |
| 8 | D05_39619438_2 | TAGTTGATTCGGAGCCAAAG | ATGTTCCGCTTATCCATCTG |
| 9 | D05_39621590_4 | CATCTAGCAAGTTAGGGTCC | GACCAATGTGTATTCGCTTG |
| 10 | D05_39630182_4 | CCTTGAGGTTGAACGAAC | ATGGCATACTGTCTCAAAGG |
| 11 | D05_39650410_3 | CAAAAGCATTGAGAGTGAGC | ATTCCTTTCCGACTTTAGCT |
| 12 | D05_39656557_5 | TTAAAATTTCATTCGGCCCC | CAGGGACAAAGCCAAAATTT |
| 13 | D05_39657245_5 | TAAATCAGTTCAACCAGCCA | GTGCTGTGGTTCCTAAGTTA |
| 14 | D05_39684635_4 | AATGGCATGTACTTAACGAG | CCATCCAAGCATGTCTAGAT |
| 15 | D05_39760189_4 | AGCCTTCTTTGAGATCCTTG | TAAAGCTATCGGGGTCCTTA |
| 16 | D05_39766367_5 | CTAGCTATTTCACGTAGGGG | TTCAACTCGACTCAAACTTG |
| 17 | D05_39789249_5 | CCCGTGCAATACAATGATTT | ATCGACCTAGATTTGATGGC |
| 18 | D05_40007094_2 | GGAATTTGGACGAATCTATC | GCCAGTTAAGCTAAAGTATGAC |
| 19 | D05_40311270_2 | GTGGTCGGTTATTATAGGCT | ACATGCAGTAGACGATCAAG |
| 20 | D05_40659196_3 | TCGGCACCATAGAAATGTTT | CTTCTTCTTACTCGTGCGTA |
| 21 | D05_40772828_4 | GCTCGATTTAGGGATGACAT | TGCTGACATTTTGGAAGGAT |
| 22 | D05_40803989_2 | CGAGTCATGAGTCCTTTAGG | TTAACGATGGTGGAGAGTTG |
| 23 | D05_40780311_5 | AATTCGGTTGCTGCTTAAAG | CCAAGGGAGATGTAGGAGTA |
| 24 | D05_37618302_2 | CGCTTCATTGTGGATAGAGA | AGTGATTCAACTTAGGCCTG |
| 25 | D05_37685795_3 | TAATGCCGCCGAACATATTA | TCCATCGTGTTGACTTGTAA |
| 26 | D05_37700794_2 | GGTTTAGTCCTTGCACCTTA | CAAGGGTGACGAAAATTACG |
| 27 | D05_37722822_3 | GCGTGTTACAGTGTGTTTAT | TAAGCTCCCCTAACCTATGT |
| 28 | D05_39595463_4 | TTGCCGGTTCGATGTATTTA | CAATTGGATCATGCTCAAGT |
| 29 | D05_39767538_3 | CAACTAAGGGCATGACAGAT | ACATCAGATCCCCTAGAACA |
| 30 | D05_39770976_2 | GGTATCAACTGAAGGCAAGA | CGTGTTGATTCATCTCTCCT |
| 31 | D05_39799227_4 | TTGTCCAAACTCTCTCAAGG | CACCCGTAATAGCATTACCA |
| 32 | D05_40008039_2 | TTCACCTTGCCTTGATACAA | CATGATATGCACGATGTTGA |
| 33 | D05_40014316_2 | TACAACCAGACATCACCCTA | TTTTGAGCTTAACCCATTGC |
| 34 | D05_40192944_5 | TCAAAGCCATGGGAGTTATC | GATGTGATATGAAAGGTGAC |
| 35 | D05_40398511_2 | CCTGCTTAATCTCAATCCGA | TCGATGTGTACTATGAGCAA |
| 36 | D05_40370515_3 | TTGTCTCGCTCAATGTACAA | GGAAATCTGCCAAACAACAA |
| 37 | D05_37672051_4 | GGGGTTAGGGGAAATAGTTC | AAGAAAAGTGAGAGTGAGTG |
| 38 | D05_40135504_2 | GGAAGCAGCTTTAGAAGGAT | GACACCCAATACTGTTACGA |
| 39 | D05_40157037_5 | TCAACCCCTAAATCAAACCC | TCTCAAGGTTTAGGGTTTGG |
| 40 | D05_40174863_2 | CATAGCATGTTAGGTGTATG | CACACCAAAACACAACTTCA |
| 41 | D05_40391275_2 | AATATTGTCGAGGGATGGTG | TTTGCCCCATTTTATTCCCT |
| 42 | D05_40781239_2 | GCAAGGAGTCTTTAAGGTGA | TTTTATCCCATCGAACTCCC |
